# Editorial: Host immune response and protective immune responses during filarial infections

**DOI:** 10.3389/fimmu.2022.1102121

**Published:** 2022-12-13

**Authors:** Manuel Ritter, Marc P. Hübner

**Affiliations:** ^1^ Institute for Medical Microbiology, Immunology and Parasitology (IMMIP), University Hospital Bonn (UKB), Bonn, Germany; ^2^ German Center for Infection Research (DZIF), partner site Bonn-Cologne, Bonn, Germany

**Keywords:** *Litomosoides sigmodontis*, filaria, innate lymphoid cell (ILC), microfilariae, vaccination, lymphatic filariasis (LF), influenza, diabetes

Filarial nematodes can cause the debilitating neglected tropical diseases onchocerciasis and lymphatic filariasis. Those diseases can lead to blindness or severe dermatitis (onchocerciasis) or lymphedema in the extremities and hydrocele in the male scrotum (lymphatic filariasis). In contrast, filaria l infections with *Mansonella* spp. and *Loa loa* are often asymptomatic without distinct clinical symptoms. Indeed, filariae actively modulate the immune system of their hosts and induce, if successful, a regulatory and anti-inflammatory milieu, which enables filarial long-term survival and limits filarial pathology. Within this special issue, a selection of articles that focus on the immune responses during filarial infection and consequences of filarial-driven immunomodulation is presented. Five of those studies used the *Litomosoides sigmodontis* mouse model of filariasis, a parasite which is regularly used as surrogate model for human-pathogenic filariae, given that it completes its life cycle in susceptible BALB/c mice with the development of the filarial progeny, the microfilariae (1). Remion et al. used the *L. sigmodontis* mouse model to demonstrate that type 2 immune responses are not only involved in protective responses to the filariae, but also in the development of filarial pathology. Their results indicate that microfilariae induce local pathology in the pleural cavity, the site of adult worm residence. Absence of type 2 immune responses in IL-4Rα/IL-5 deficient mice drastically reduced this pleural cavity pathology, despite significantly higher microfilariae numbers. Interestingly, absence of IL-4Rα/IL-5 signaling also altered the phenotype of pleural macrophages, leading to changes in the arginine metabolic pathway and failure of maturation into resident F4/80^high^ large macrophages. Next to macrophages, additional innate immune cells were investigated during filarial infection by the present collection, namely innate lymphoid cell types. Reichwald et al. show that type 2 innate lymphoid cells (ILC2s) and the associated type 2 cytokines IL-5 and IL-13 expand during *L. sigmodontis* infection to a stronger extend in semi-susceptible C57BL/6 mice, which clear the infection shortly after the development into adult worms, in comparison to susceptible BALB/c mice. The study provides first indications that ILC2s are involved in protective immune responses during filarial infection, as depletion of ILC2s in T cell and B cell-deficient RAG2 C57BL/6 mice lead to an increase in microfilariae numbers. A possible interaction of type 1 and type 2 innate immune responses during filarial infection is provided by Pionnier et al., showing that NKp46+ natural killer (NK) innate lymphoid cells contribute to protective immune responses against filariae. NKp46+ NK cells expand during the first week of experimental *Brugia malayi* infection of mice and depletion of this cell population in RAG2-deficient C57BL/6 mice led to an increased adult worm recovery. Interestingly, this was associated with an impaired eosinophil and neutrophil recruitment, two cell types which are well-known for mediating protective immune responses against filariae (Ehrens et al.) (2). Further research to decipher the role of specific cell types during filarial infection is likely to follow, as Wiszniewsky et al. established an adaptive transfer model using RAG2IL-2Rγ-deficient C57BL/6 mice which lack T, B and NK cells resulting in long-term patent *L. sigmodontis* infections in comparison to semi-susceptible C57BL/6 and susceptible BALB/c wildtype mice. The authors show that, in contrast to CD8+ T cells, adoptive transfer of CD4+ T cells isolated from acute *L. sigmodontis*-infected C57BL/6 donor mice or mice that already cleared the infection, efficiently eliminate the parasites, prevent inflammation at the site of infection and reduce filarial embryogenesis in RAG2IL-2Rγ-deficient recipients. In addition, the parasite clearance was associated with Th17 polarization of the CD4+ T cells. Filariae-induced immune responses do not only affect parasite survival and development of pathology, but they also alter bystander responses and therefore the outcome of co-infections and vaccination efficacy. In this regard, Hardisty et al. demonstrate that the impact of filarial infections on a H1N1 influenza A infection is dependent on the filarial life cycle stage. Patent *L. sigmodontis* infection resulted in significantly higher viral titers, increased weight loss and clinical signs, whereas an acute filarial infection did neither worsen the clinical symptoms nor alter the viral load. The study by Stetter et al. used a similar *L. sigmodontis* H1N1 influenza A mouse model to investigate the impact of anthelmintic treatment on vaccination efficacy. Clearance of the filarial infection with flubendazole immediately before or two weeks before the anti-influenza vaccination failed to protect mice from challenge infection with the 2009 pH1N1 influenza A strain. Despite the drug-induced worm clearance, expanded type 1 regulatory T cell populations were maintained, which indicates persistent immunosuppression of vaccine responses. Thus, additional boosters may be required for effective vaccination in people that have present or past helminth infections. A proof-of-concept for such a vaccine booster was provided in the study by Stetter et al., which resulted in full protection. Finally, the present collection includes a publication that addresses the impact of diabetes on immune responses in lymphatic filariasis patients. This is of importance, as prevalence of diabetes is globally increasing, especially in low and middle income countries including countries co-endemic for filariae. Several studies showed in the past that helminth infections may protect against metabolic and autoimmune diseases, including type 1 and type 2 diabetes (3). However, the impact of diabetes on immune responses in filariasis patients is less well studied and important, as e.g. increased inflammatory and reduced regulatory immune responses are associated with the development of pathology in lymphatic filariasis patients (4). The study of Sibi et al. demonstrates that lymphatic filariasis patients suffering of diabetes have stronger Toll-like receptor and filarial-specific immune responses in comparison to non-diabetic filariasis patients, indicating that diabetes may present a risk factor to develop lymphatic filariasis pathology. Taken together, this collection enhances our understanding of host immune responses during filarial infection, including protective immune responses against filariae, impact on co-infections as well as vaccines.

## Author contributions

Invited the authors for the collection, handled the review process and wrote the editorial: MR, MH. All authors contributed to the article and approved the submitted version.
